# Neglected Rectal Examination

**Published:** 1917-12

**Authors:** James A. McVeigh

**Affiliations:** Detroit, Mich.


### NEGLECTED RECTAL EXAMINATIONS.

**By James A. McVeigh, M.D., Detroit, Mich.**

A thorough rectal examimnation is a source of comfort to the patient and satisfaction to the doctor. It enables the latter to make a correct diagnosis and advise proper treatment.

Rectal examinations are not difficult and should be ocular, digital and instrumental. The physician should never accept the patient's diagnosis. This is not infrequently done and such an unscientific procedure is usually productive of unsatisfactory result.

Indifference and carelessness on the part of members of the medicad profession in conducting rectal examinations being repidly lessened, owing largely to the influence of the American Proctologic Society. The remedy lies in persistently reminding the men engaged in general practice of the necessity of making thorough rectal examinations whenever indicated, and the men who are following this special line of work are the ones who must be most active in conducting this campaign of education.

